# Processing of visual signals related to self-motion in the cerebellum of pigeons

**DOI:** 10.3389/fnbeh.2013.00004

**Published:** 2013-02-12

**Authors:** Douglas R. Wylie

**Affiliations:** Centre for Neuroscience and Department of Psychology, University of AlbertaEdmonton, AB, Canada

**Keywords:** optic flow, cerebellum, vestibulocerebellum, zebrin, accessory optic system, pretectum, oculomotor cerebellum

## Abstract

In this paper I describe the key features of optic flow processing in pigeons. Optic flow is the visual motion that occurs across the entire retina as a result of self-motion and is processed by subcortical visual pathways that project to the cerebellum. These pathways originate in two retinal-recipient nuclei, the nucleus of the basal optic root (nBOR) and the nucleus lentiformis mesencephali, which project to the vestibulocerebellum (VbC) (folia IXcd and X), directly as mossy fibers, and indirectly as climbing fibers from the inferior olive. Optic flow information is integrated with vestibular input in the VbC. There is a clear separation of function in the VbC: Purkinje cells in the flocculus process optic flow resulting from self-rotation, whereas Purkinje cells in the uvula/nodulus process optic flow resulting from self-translation. Furthermore, Purkinje cells with particular optic flow preferences are organized topographically into parasagittal “zones.” These zones are correlated with expression of the isoenzyme aldolase C, also known as zebrin II (ZII). ZII expression is heterogeneous such that there are parasagittal stripes of Purkinje cells that have high expression (ZII+) alternating with stripes of Purkinje cells with low expression (ZII−). A functional zone spans a ZII± stripe pair. That is, each zone that contains Purkinje cells responsive to a particular pattern of optic flow is subdivided into a strip containing ZII+ Purkinje cells and a strip containing ZII− Purkinje cells. Additionally, there is optic flow input to folia VI–VIII of the cerebellum from lentiformis mesencephali. These folia also receive visual input from the tectofugal system via pontine nuclei. As the tectofugal system is involved in the analysis of local motion, there is integration of optic flow and local motion information in VI–VIII. This part of the cerebellum may be important for moving through a cluttered environment.

## Introduction

As an observer moves through an environment consisting of numerous objects and surfaces, visual motion occurs across the entire retina. This is known as “optic flow” (Gibson, 1954). The processing of optic flow is important for numerous behaviors and processes including perception of self-motion, the control of posture and locomotion, and navigation (Waespe and Henn, 1987; Srinivasan et al., 1996; Warren et al., 2001; Kearns et al., 2002). Although research has shown that the cortical area MST is important for the analysis of optic flow in primates (e.g., Duffy and Wurtz, 1991; for review see Duffy, 2004), there is a much older literature showing that the terminal nuclei of the accessory optic system (AOS) and the nucleus of the optic tract in the pretectum process optic flow (Simpson and Alley, 1974; Collewijn, 1975; for reviews see Simpson, 1984; Gamlin, 2005; Giolli et al., 2005). The AOS and pretectum are found in all vertebrate classes (Fite, 1985; McKenna and Wallman, 1985; Weber, 1985) and are highly conserved with respect to physiological response properties and neuroanatomical connections (Ibbotson and Price, 2001; Voogd and Wylie, 2004).

In birds, optic flow analysis begins with two retinal recipient nuclei: the nucleus of the basal optic root (nBOR; homologous to the terminal nuclei in mammals) of the AOS, and the pretectal nucleus lentiformis mesencephali (LM; homologous to the nucleus of the optic tract). Retinal input to nBOR arises from a distinct subset of ganglion cells; “displaced” ganglion cells, so called because they are found in the inner plexiform layer rather than the ganglion cell layer (Karten et al., 1977; Reiner et al., 1979; Fite et al., 1981). The connections of LM and nBOR are extensive and include structures involved in axial motor control, oculomotor control, and nuclei in other visual pathways (Clarke, 1977; Brecha et al., 1980; Gamlin and Cohen, 1988; Wild, 1989; Wylie et al., 1997, 1998b). This review will focus on my work describing how optic flow information is processed en route to, and within, the cerebellum of pigeons although I note similarities and differences with other species. There are several reasons why pigeons are the subjects of this research. In addition to practical considerations such as expense, availability, and manageability, pigeons are especially useful for studying optic flow processing in the cerebellum for several reasons. The avian and mammalian visual pathways are very similar with respect to anatomical and functional organization (Karten and Shimizu, 1991; Nguyen et al., 2004). This is particularly true for the visual-cerebellar pathways involved in processing optic (Voogd and Wylie, 2004; see below). Furthermore, birds in general have a highly developed cerebellum (Larsell, 1967) which is easily accessible for electrophysiological and anatomical study. Finally, pigeons are a diurnal species, and as creatures of flight, the analysis of optic flow is critical to their survival.

**Figure 1:**
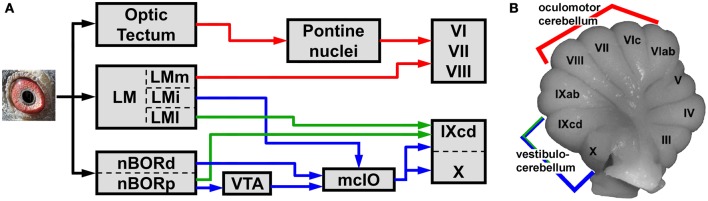
**A reduced schematic, showing the visual pathways (A) to the cerebellum (B) in birds.** The cerebellum is divided into folia, numbered I–X from anterior to posterior (Larsell, 1967). Folia IXcd and X comprise the vestibulocerebellum, which receives optic flow input from the nucleus of the basal optic root (nBOR) and lentiformis mesencephali (LM) via a climbing fiber pathway through the medial column of the inferior olive (mcIO) (blue pathway) (Arends and Voogd, 1989; Lau et al., 1998). The LM and nBOR also project directly to folium IXcd as mossy fibers (green pathways) (Brauth and Karten, 1977; Clarke, 1977; Brecha et al., 1980; Wylie and Linkenhoker, 1996). The LM also projects to folia VI–VIII (red pathway), which are part of the oculomotor cerebellum (Voogd and Barmack, 2006). These folia also receive visual motion signals from a tecto-pontine system (red pathway) (Reiner and Karten, 1982). See text for additional details. LMm, i, l: the medial, intercalatus, and lateral divisions of LM. nBORd, p: the dorsalis and proper divisions of nBOR. VTA: ventral tegmental area.

The pathways from LM and nBOR to the cerebellum are shown in Figure 1. First, LM and nBOR project to the medial column of the inferior olive (mcIO), which in turn provides climbing fiber input to the vestibulocerebellum (VbC; folia IXcd and X) (blue pathway) (Clarke, 1977; Brecha et al., 1980; Gamlin and Cohen, 1988; Arends and Voogd, 1989; Wylie et al., 1997, 1999, 2007, 2008; Lau et al., 1998; Crowder et al., 2000; Wylie, 2001; Winship and Wylie, 2003; Pakan et al., 2005, 2006, 2010; Pakan and Wylie, 2006, 2008; Winship et al., 2006; Iwaniuk et al., 2009). Second, LM and nBOR project directly to IXcd of the VbC as mossy fibers (Brauth and Karten, 1977; Gamlin and Cohen, 1988; Wylie and Linkenhoker, 1996; Pakan et al., 2006, 2010; Wylie et al., 2007, 2008; Iwaniuk et al., 2009). Third, LM projects to folia VI–VIII, an area known as the “oculomotor cerebellum” (for review see Voogd and Barmack, 2006), where there is interaction with local motion inputs from a tecto-pontine system (Pakan et al., 2006). Each of the pathways is discussed below.

## Motion processing in the nucleus of the basal optic root and lentiformis mesencephali

**Figure 2:**
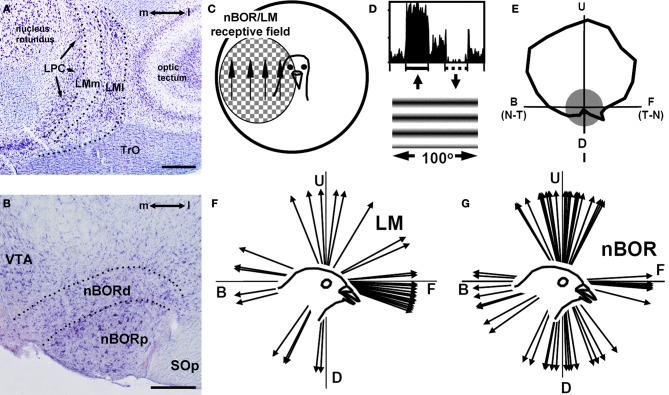
**Basic visual processing in the accessory optic system in birds. (A)** and **(B)**, Respectively, show Nissl-stained coronal sections through the pigeon lentiformis mesenceophali (LM) and nucleus of the basal optic root (nBOR). **(C)** Indicates that most nBOR and LM neurons have large receptive fields in the contralateral visual field and exhibit directional tuning in response to largefield motion (e.g., Burns and Wallman, 1981; Winterson and Brauth, 1985). **(D)** Shows the response of a nBOR neuron to upward (excitation) and downward (inhibition) motion of a large drifting sine-wave grating (from Crowder and Wylie, 2001). **(E)** Shows a directional tuning curve of a typical nBOR neuron. Firing rate (spikes/s) is plotted as a function of the direction of motion in polar coordinates, and the gray circle represents the neuron's spontaneous firing rate. The directions are indicated as follows: U, upward; D, downward; F, forward or temporal-to-nasal (T-N), and B, backward or nasal-to-temporal (N-T). **(F)** Shows a distribution of the direction preferences of LM neurons in pigeons: most prefer forward motion (adapted from Wylie and Crowder, 2000). **(G)** Shows a distribution of the direction preferences of nBOR neurons in pigeons: most prefer upward, downward, or backward motion (Crowder et al., 2003).

As self-motion causes visual motion across the entire retina, one would expect a system that analyzes this optic flow would respond to motion over large parts of the retina. Indeed, LM (Figure 2A) and nBOR (Figure 2B) neurons have large contralateral receptive fields (Figure 2C) averaging 60° in diameter with the largest encompassing the entire monocular visual field. These neurons are directionally selective in response to large stimuli, such as random dot patterns, checkerboards, and gratings (Figure 2D) (Burns and Wallman, 1981; Morgan and Frost, 1981; Gioanni et al., 1984). A tuning curve for a nBOR neuron is shown in Figure 2E (Wylie and Frost, 1990a). Although broadly tuned, the neuron shows a maximal response to upward motion (preferred direction) and is inhibited by downward motion (anti-preferred direction). Neurons in nBOR and LM show a complementary pattern of direction selectivity. In LM, most (>50%) neurons prefer forward (i.e., temporal-to-nasal) motion (Figure 2F) (Winterson and Brauth, 1985; Wylie and Frost, 1996; Wylie and Crowder, 2000). In contrast, neurons preferring upward, downward and backward (i.e., nasal-to-temporal) motion are about equally abundant in nBOR, but fewer (5–10%) prefer forward motion (Figure 2G) (Gioanni et al., 1984; Wylie and Frost, 1990a; Crowder et al., 2003).

## Distinguishing self-translation and self-rotation in the vestibulocerebellum (VBC)

**Figure 3:**
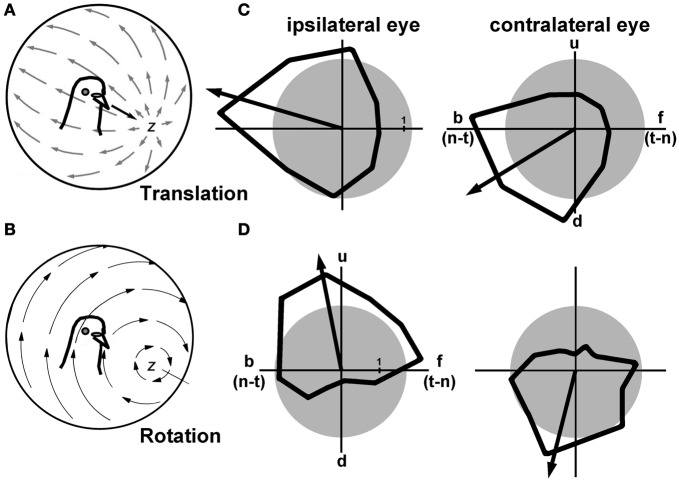
**(A)** Shows the pattern of optic flow resulting from forward translation along the z-axis, as projected onto a sphere surrounding the bird. The arrows represent local image motion in the flowfield. **(B)** Shows the optic flow resulting from rotation about the z-axis (roll). **(C)** and **(D)** Show the directional tuning curves in response to large-field stimulation of the ipsi- and contralateral eyes for Purkinje cells in the vestibulocerebellum. The arrows represent the peak best fit sine wave to the tuning curve, and serves as a proxy for the preferred direction. The cell in **(C)** preferred backward (b) [i.e., nasal-to-temporal (n-t)] motion in both eyes, which would result from forward self-translation (adapted from Graham and Wylie, 2012). The cell in **(D)** preferred upward (u) motion in the ipsilateral eye, and downward motion in the contralateral eye, which results from rotation of the head about the z-axis (roll) (adapted from Wylie and Frost, 1991). The gray circles represent the spontaneous firing rates of these neurons, which is typically about 1 spikes/s. d: downward motion; f: forward motion [i.e., temporal-to-nasal (t-n)].

The motion of any object through 3-dimensional space can be described with reference to its translation between two points, and its rotation about an intrinsic axis. This can also be applied to self-motion of an organism, and vertebrates do have mechanisms to detect both self-translation and self-rotation. The vestibular system consists of the semicircular canals, which detect head rotation, and the otolith organs, which detect head acceleration resulting from gravity and self-translation (Wilson and Melvill Jones, 1979). A neural system involved in analyzing optic flow can also encode self-translation and self-rotation. The patterns of optic flow resulting from self-translation and self-rotation are quite different. Figures 3A and B show, respectively, the patterns of optic flow resulting from translation along, and rotation about, the *z*-axis. These are shown as projected onto imaginary spheres surrounding the animal, where the arrows indicate local motion within the flowfield (Gibson, 1954). Assuming no eye movements, during self-translation there is a focus of expansion in the direction of self-motion, and backward motion along the equator of this sphere in both visual fields (Figure 3A). Not visible in the figure, there would also be a focus of contraction behind the animal's head. For self-rotation about the *z*-axis, there is circular motion about the axis of rotation, but along the equator of this sphere there is upward and downward motion in the right and left visual fields respectively. Although the neurons in LM and nBOR have large receptive fields for analyzing optic flow, they cannot distinguish optic flow patterns resulting from self-rotation and self-translation. For example, a neuron preferring upward motion, such as that depicted in Figures 2C–E, would respond equally well to downward-translation and a rightward roll of the head. For a predominantly lateral-eyed animal such as a pigeon, a simple solution is to integrate information from the ipsi- and contralateral visual fields. This is what occurs in the olivo-vestibulocerebellar pathway shown in blue in Figure 1. In Figures 3C and D, examples are shown from the VbC on the left side of the brain, where directional tuning to largefield moving stimuli was measured for both the ipsilateral and contralateral eyes. The neuron in Figure 3C responded best to backward (nasal-to-temporal) motion in both eyes, which would result from forward self-translation. The neuron in Figure 3D responded best to upward motion in the ipsilateral eye, and downward motion in the contralateral eye, which would result from a rightward rotation about the *z*-axis (roll). Although there are a few neurons in nBOR, LM, and the ventral tegmental area (VTA) that have such binocular receptive fields that respond to particular patterns of optic flow resulting from self-translation and self-rotation (Wylie and Frost, 1990b, 1999b; Wylie, 2000), almost all neurons in mcIO and the VbC have panoramic receptive fields (Wylie and Frost, 1991, 1993, 1999a; Wylie et al., 1993; Winship and Wylie, 2001). Moreover there is a clear topographic organization of neurons responsive to translational and rotational optic flow (Winship and Wylie, 2001; Pakan et al., 2005; Graham and Wylie, 2012).

The pathway from the nBOR and LM to the VbC is as follows. The mcIO receives a projection from the ipsilateral LM (Clarke, 1977). This projection is mainly directed to the caudal mcIO (Wylie, 2001; Pakan et al., 2010) and arises from a distinct group of medium-sized fusiform cells found in a thin strip along the border of the medial and lateral subnuclei of LM (LMm, LMl) (Gamlin and Cohen, 1988; Wylie, 2001; Pakan et al., 2006). We have referred to this region as the intercalated nucleus of LM (LMi). The mcIO also receives a bilateral input from nBOR. It is directed mainly to the rostral mcIO (Wylie, 2001; Pakan et al., 2010) and arises from small neurons in nBOR dorsalis (nBORd) and the adjacent VTA (Brecha et al., 1980; Wylie, 2001; Pakan et al., 2006). The mcIO projects to the VbC (folia IXcd and X) as climbing fibers. This projection is topographic such that the medial mcIO projects to the lateral VbC and the lateral mcIO projects to the medial VbC (Arends and Voogd, 1989; Lau et al., 1998; Wylie et al., 1999; Crowder et al., 2000). The lateral VbC is known as the flocculus, whereas the medial VbC is the uvula/nodulus.

## Encoding of rotational optic flow

Generally, one describes the rotation of an object in space with reference to its component rotations about three orthogonal axes: roll (*z*), pitch (*x*) and yaw (*y*). As outlined in this section, a three-axis reference frame underlies the analysis of rotational optic flow. These axes are orthogonal, but they are not roll, pitch, and yaw.

**Figure 4:**
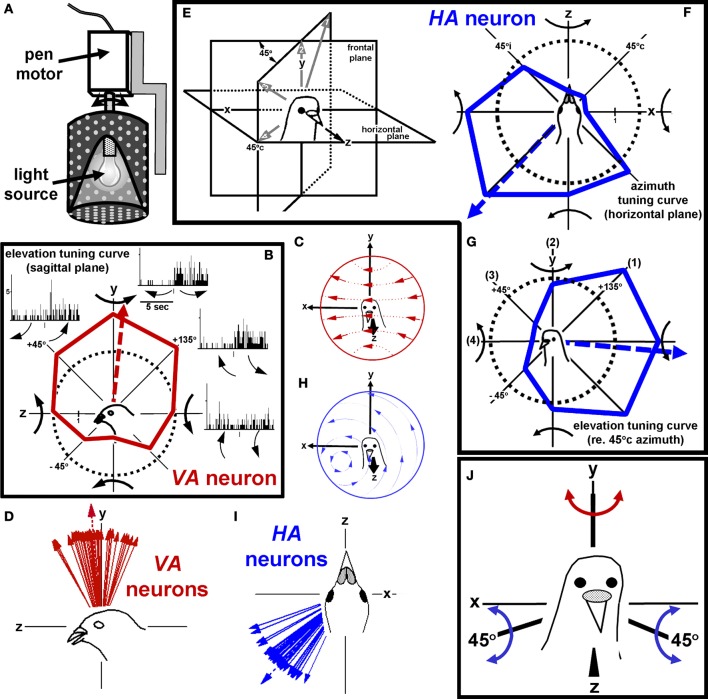
**Stimulating rotation-sensitive optic flow neurons in the pigeon vestibulocerebellum (VbC). (A)** Shows a schematic of the planetarium projector used to simulate rotational optic flow. **(B)** Shows the elevation tuning curve for a vertical axis (*VA*) neuron. The flowfield that maximally stimulates *VA* neurons shown in **(C)**, and the best axes of several *VA* neurons are shown in **(D)**. **(E–G)** Shows axis tuning for an *HA* neuron. **(F)** Shows an azimuth tuning curve plotting the responses to rotation about axes in the horizontal (*xz*) plane. **(G)** Shows an azimuth tuning curve in a vertical plane that intersects the horizontal plane through 45° contralateral azimuth (45°c). The flowfield that maximally stimulates *HA* neurons in shown in **(H)**, and the best axes of several *HA* neurons are shown in **(I)**. **(J)** Shows the reference frame for rotational optic flow responses in the VbC. Considering both sides of the brain, it consists of three orthogonal axes: the vertical (*y*) axis and two horizontal axes oriented 45° to the midline. All responses in this and subsequent figures refer to recording from neurons in the VbC on the left side of the brain. These data are from Wylie and Frost (1993). See text for additional details.

To provide an effective stimulus for neurons responsive to rotational optic flow in the flocculus of rabbits, Jerry Simpson and Werner Graf designed a planetarium projector, which projected a flowfield onto the floor, walls and ceiling of the room (Simpson et al., 1981, 1988). The projector was suspended in gimbals, such that axis of rotation could be aligned to any orientation within 3-dimensional space. We used a similar device, depicted in Figure 4A, to stimulate the complex spike activity of Purkinje cells in the pigeon flocculus. Our findings (Wylie and Frost, 1993) were essentially identical to those of Graf et al. (1988). In the flocculus, there are two types of neurons: one prefers rotational optic flow about the vertical (*y*) axis (*VA* neurons) and the other prefers rotational optic flow about an horizontal axis oriented 45° to the midline (*HA* neurons). Figure 4B shows the responses of a *VA* neuron in the left flocculus to rotational optic flow about four axes in the sagittal (*yz*) plane. Each peri-stimulus time histogram (PSTH) is summed from 10 sweeps, where each sweep consisted of 5 s of rotation in one direction followed by 5 s of rotation in the opposite direction. An elevation tuning curve is also shown, where the firing rate (solid red line) is plotted as a function of the axis of rotation. The broken circle represents the spontaneous rate, and the broken red line indicates the preferred axis as calculated from the best fit sine wave. The direction of each curved arrow represents the direction of head motion that would cause the presented flowfield. Thus, the cell responds best to leftward rotation of the head about the vertical (*y*) axis. The flowfield that maximally stimulates *VA* neurons in shown in Figure 4C, and the best axes of several *VA* neurons are shown in Figure 4D. The largest arrow with the broken shaft represents the mean of the distribution. Figures 4E–G shows axis tuning for an *HA* neuron. Figure 4F shows the azimuth tuning curve plotting the responses to rotation about axes in the horizontal (*xz*) plane, whereas Figure 4G shows the azimuth tuning curve in a vertical plane that intersects the horizontal plane through 45° contralateral azimuth (45°c) for the same neurons. This vertical plane is depicted in Figure 4E, and the axes numbered 1–4 in Figure 4E correspond to those in Figure 4G. This neuron responded best to rotation about an horizontal axis oriented at 45°c/135°i azimuth. The flowfield that maximally stimulates *HA* neurons in shown in Figure 4H, and the best axes of several *HA* neurons are shown in Figure 4I.

Figure 4J shows the reference frame of rotational optic flow neurons considering neurons on both sides of the brain. It consists of three orthogonal axis: the vertical axis, and two horizontal axes oriented 45° to the midline. Simpson, Graf and colleagues noted that this is the same reference frame as the vestibular semicircular canals, and the eye muscles (Simpson et al., 1979, 1981, 1988; Ezure and Graf, 1984; Simpson and Graf, 1985; Graf et al., 1988; see also Wylie and Frost, 1996). The horizontal canals are maximally responsive to rotation about the vertical axis, whereas one anterior canal (and the contralateral coplanar posterior canal) responds best to rotation about a horizontal axis oriented 45° to the midline. With respect to the eye muscles, the horizontal recti rotate the eyes about the vertical axis, whereas the vertical recti and oblique muscles rotate the eyes about an horizontal axis oriented at 45° to the midline. Thus, the sensory systems involved in analysis of self-rotation (vestibular and optic flow) and the output of this system (i.e., the eye muscles which generate compensatory rotary eye movements) all share the same spatial reference frame.

## Encoding of translational optic flow

**Figure 5:**
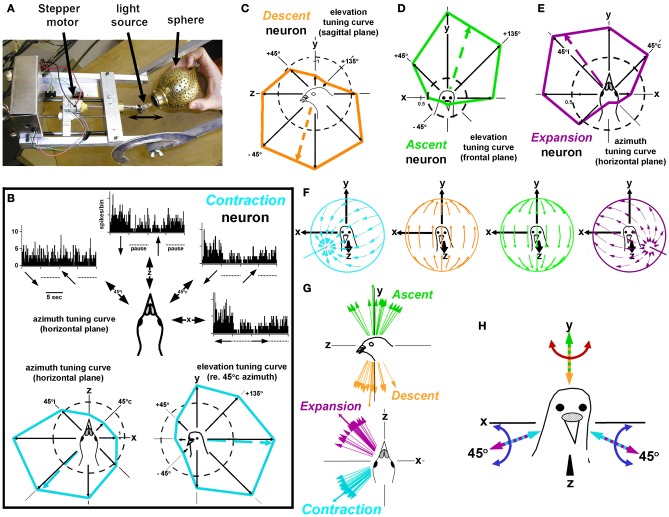
**Stimulating translation-sensitive optic flow neurons in the pigeon vestibulocerebellum (VbC). (A)** Shows a schematic of the translator projector used to simulate translational optic flow. This was suspended above the bird's head in gimbals such that the axis of translation could be oriented anywhere in 3-dimensional space. **(B)** Shows the responses of a *Contraction* neuron. An azimuth tuning curve (*xz* plane) is shown as well as an elevation tuning curve in a vertical plane that intersects the horizontal plane at 45°c azimuth. **(C–E)** Show tuning curves for the other three types of translation neurons in the VbC: *Descent*, *Ascent* and *Expansion*. The flowfields that maximally stimulate each of the four types are shown in **(F)**, and the best axes of translation for the four groups are shown in **(G)**. **(H)** Shows the common reference frame for translational and rotational optic flow reponses in the VbC. The data are from Wylie and Frost (1999b). See text for additional details.

To simulate translational optic flow we designed the device shown in Figure 5A, which projected panoramic translational optic flow on to the walls, ceiling, and floor of the room, and we recorded from Purkinje cells in the uvula/nodulus in pigeons (Wylie et al., 1998a; Wylie and Frost, 1999a). There are four types of optic flow neurons in the uvula/nodulus: *Contraction*, *Expansion*, *Ascent*, and *Descent*. Figure 5B shows the responses of a *Contraction* neuron in the left uvula/nodulus in response to translational optic flow along several axes. PSTHs show the responses to translational optic flow along 4 axes in the horizontal (*xz*) plane. Each PSTH is summed from 20 sweeps, where each sweep consisted of 5 s of translation in one direction followed by a 5 s pause, then 5 s of motion in the opposite direction followed by a 5 s pause. An azimuth tuning curve (*xz* plane) is shown as well as an elevation tuning curve in a vertical plane that intersects the horizontal place at 45°c azimuth. The direction of each arrow represents the direction of head motion that would cause the presented flowfield. This cell responds best to backward translation along an horizontal axis oriented at 45°c/135°i azimuth. This results in a flowfield with a focus of contraction at 45°c azimuth. Figures 5C–E shows tuning curves for the other three types of translation neurons in the VbC: *Descent*, *Ascent* and *Expansion*. The flowfields that maximally stimulate each of the four types are shown in Figure 5F, and the best axes of translation for the four groups are shown in Figure 5G. Figure 5H shows the common reference frame for translational and rotational optic flow responses in the VbC. Considering both sides of the brain, the reference frame consists of three orthogonal axes: the vertical (*y*) axis and two horizontal axes oriented 45° to the midline. Note that this is the same reference frame as that of the rotational optic flow system in the flocculus. We have previously argued how this reference frame is optimal on several accounts (see Frost and Wylie, 2000).

Although the processing of rotational optic flow in the flocculus is essentially identical in pigeons and rabbits (see previous section), the same cannot be said for the uvula/nodulus. In the uvula/nodulus of rabbits, *VA* and *HA* neurons are found (Kano et al., 1990; Wylie et al., 1994) in addition to some Purkinje cells where the complex spike activity is modulated by vestibular stimulation originating in the otolith organs and vestibular canals (Barmack and Shojaku, 1992). Purkinje cell complex spike activity responsive to translational optic flow has yet to be observed in any species but the pigeon. However, Yakusheva et al. (2008) showed that simple spike activity of Purkinje cells in the uvula/nodulus in monkeys responds to self-translation. Thus is seems that the uvula/nodulus in mammals may be involved in processing both self-translation and self-rotation.

## Bipartite receptive structure of optic flow neurons in the vestibulocerbellum

**Figure 6:**
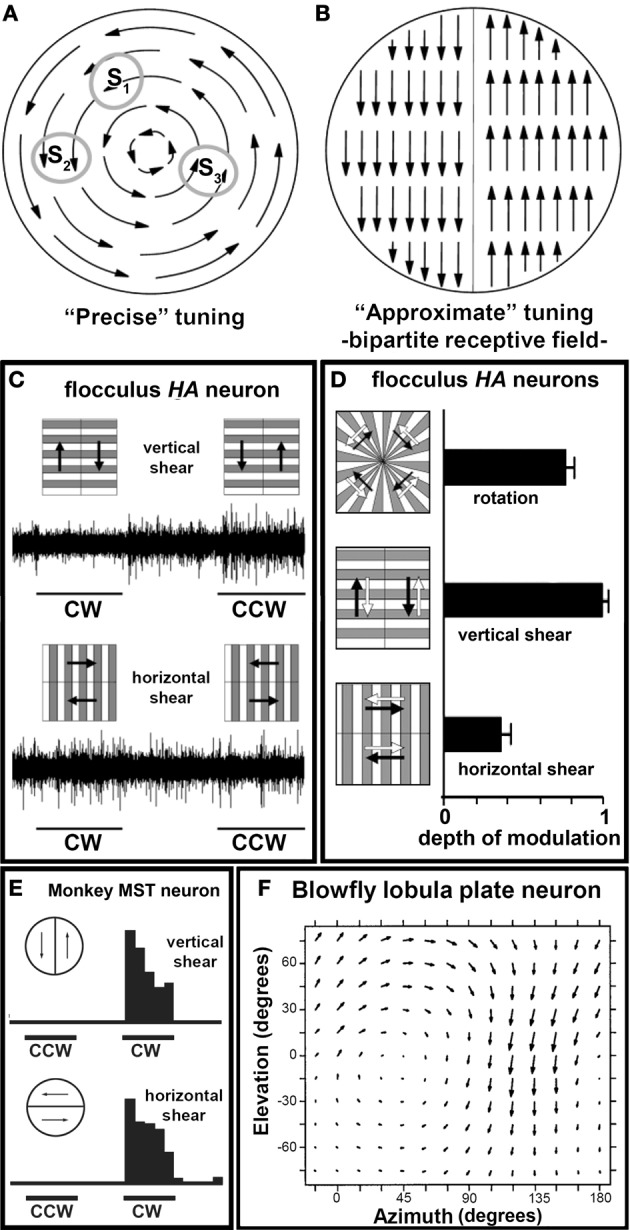
**(A)** and **(B)**, Respectively, show receptive fields either “precisely” tuned for rotational optic flow by pooling many local motion detectors with different direction preferences, or “approximately” tuned with a *bipartite* receptive field structure. **(C)** Shows the responses of an *HA* neuron to composite stimuli. The neuron responded much better to vertical shear as opposed to horizontal shear, indicating that it has a bipartite receptive field structure shown in **(B)** (Winship and Wylie, 2006). **(D)** Shows the normalized depth of modulation [(CCW−CW)/(CCW+CW)] for all rotation units (*n* = 22) in response to the three stimulus configurations illustrated (mean ± s.e.m). Note that the cells responded better to the vertical shear as opposed to the true rotation (Winship and Wylie, 2006). **(E)** Shows the responses of a rotation sensitive neuron in monkey area MST to similar stimuli. It responded equally well to vertical and horizontal shear, indicating that it has a precisely tuned receptive field (from Tanaka and Saito, 1989). **(F)** Shows the responses of an optic flow neuron in the lobula plate in blowflies to local stimulation. These neurons have an underlying receptive field with precise tuning (from Krapp et al., 1998).

Figure 6A depicts the flowfield that would result from a rightward rotation about the roll axis. To construct a receptive field sensitive to this flowfield, one could pool information from local motion detectors with predictably varying direction preferences: leftward/downward at S_1_, downward at S_2_, upward at S_3_, etc. This is not the case for the optic flow cells in the VbC. Rather they have a receptive field structure that provides a crude approximation to the preferred optic flow pattern by pooling information from two motion detectors with opposing direction preferences as illustrated in Figure 6B. Such a “bipartite” receptive field was suggested by Simpson et al. (1979, 1981, 1988) for the *HA* neurons in the rabbit flocculus. Winship and Wylie (2006) showed that the bipartitie receptive field type of arrangement underlies the receptive field structure for neurons in the pigeon flocculus and uvula/nodulus. Figure 6C shows some of the critical data for an *HA* neuron in the pigeon flocculus. The cell was stimulated with the two composite stimuli depicted. We predicted that if the receptive field was precisely tuned to rotation (as in Figure 6A), the cell would modulate equally to the “horizontal shear” and “vertical shear” conditions as an equal number of motion detectors would be excited by both stimulus configurations. However, the cell showed maximal modulation to the vertical shear configuration and no modulation to the horizontal shear condition, indicating the underlying receptive field is bipartite as indicated in Figure 6B. Data for all (*n* = 22) flocculus *HA* neurons are shown in Figure 6D. Here the normalized depth of modulation is shown in response to the vertical and horizontal shear stimuli, as well as true rotation. Note that the cells showed little modulation to the horizontal shear, and more to the vertical shear compared to rotation. Again, these data support the idea of a bipartite receptive field organization.

Optic flow neurons sensitive to translational and rotational patterns are also found in the primate cortical area MST (Duffy and Wurtz, 1991) and in the lobula plate in blowflies (Krapp and Hengstenberg, 1996), and these neurons have an underlying receptive field with precise tuning. Figure 6E shows data from a MST neuron that preferred clockwise (CW) optic flow. Note that it responded equally well to vertical and horizontal shear (Tanaka and Saito, 1989). Tanaka et al. (1989) have used other composite stimuli to stimulate MST neurons and shown that the closer the stimulus matches the preferred flowfield, the greater the response of the neuron. Figure 6F shows data from a neuron in the blowfly visual system that preferred rotational optic flow (Krapp et al., 1998). The local direction preferences within the flowfield are shown by the arrows. Thus, optic flow neurons in the MST and blowfly visual system, unlike VbC neurons, do pool information from several local motion detectors with predictable differences in direction preference to create a receptive field with precise tuning.

## Vestibular input to the vestibulocerebellum

**Figure 7:**
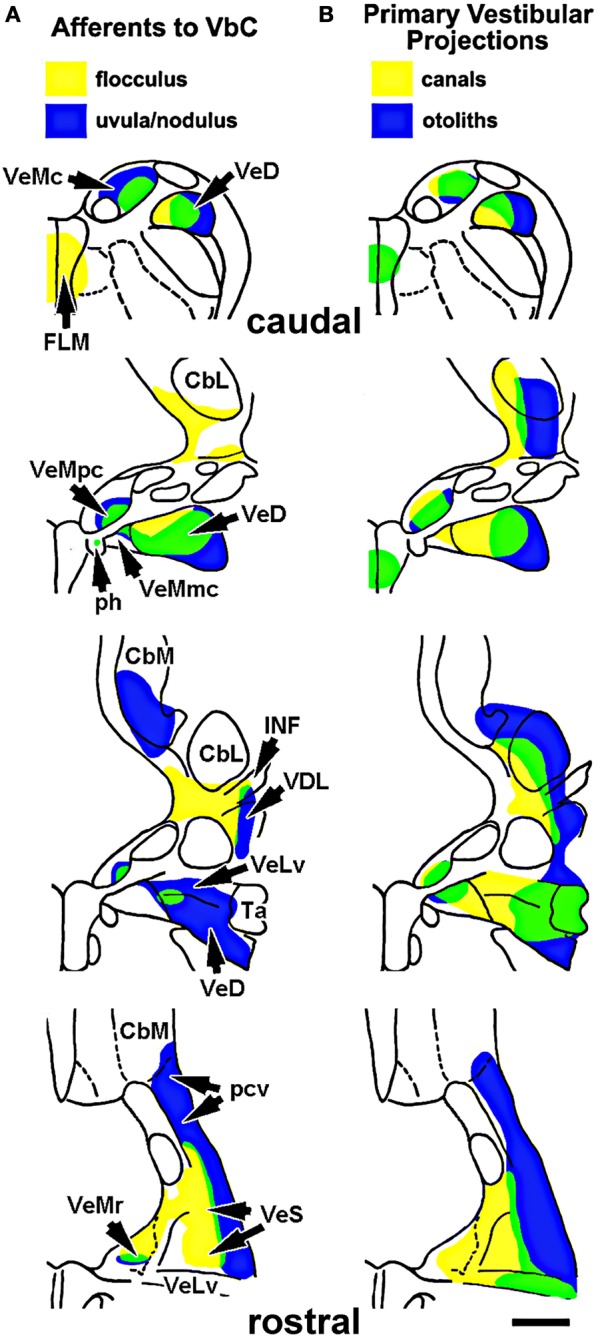
**(A)** Shows the areas of the vestibular nuclei that project to the flocculus (yellow) and uvula-nodulus (blue). Green represents areas of overlap, which contains cells that project to the flocculus and the uvula/nodulus (based on Pakan et al., 2008). **(B)** Shows the areas of the vestibular nuclei that receive input from the otolith organs (blue) and semicircular canals (yellow). The areas in green receive input from both the semicircular canals and otolith organs (based on Schwarz and Schwarz, 1983 and Dickman and Fang, 1996). Abbreviations: VeMc, r, pc, mc: the caudal, rostral, parvocellular, and magnocellular divisions of the medial vestibular nucleus; VeD: descending vestibular nucleus; FLM: medial longitudinal fasciculus; ph: prepositus hypoglossi; CbM: medial cerebellar nucleus; CbL: lateral cerebellar nucleus; INF: infracerebellar nucleus; VDL: dorsolateral vestibular nucleus; VeLv: lateral vestibular nucleus, ventral division; Ta: tangential nucleus; pcv: cerebellovestibular process. Scale bar = 1 mm.

Given that the flocculus and uvula/nodulus are involved in the processing of optic flow resulting from self-rotation and self-translation, respectively, one might expect that the flocculus would be associated with vestibular input from the semi-circular canals, whereas the uvula/nodulus would be associated with input from the otolith organs. By examining the input from the vestibular nuclei to the VbC, we have shown that this is generally the case (Pakan et al., 2008). Shown in Figure 7, we injected retrograde tracers in the flocculus and uvula/nodulus and analyzed the distribution of retrogradely labeled cells in the vestibular nuclei (Figure 7A) and compared this to descriptions of the primary vestibular afferents from the canals and otolith organs to the vestibular nuclei (Figure 7B) (Schwarz and Schwarz, 1983; Dickman and Fang, 1996). Although hardly absolute, the regions that project to the flocculus tend to receive input from the semicircular canals, whereas the regions that project to the uvula/nodulus receive input from the otolith organs. For example, in both the descending vestibular nucleus (VeD) and superior vestibular nucleus (VeS), the lateral portion receives input primarily from the otolith organs (blue in Figure 7B) and projects primarily to the uvula-nodulus (blue in Figure 7A), whereas the medial portions of VeD and VeS receive input primarily from the vestibular canals (yellow in Figure 7B) and project primarily to the flocculus (yellow in Figure 7A).

## Zonal organization of the vestibulocerebellum

**Figure 8:**
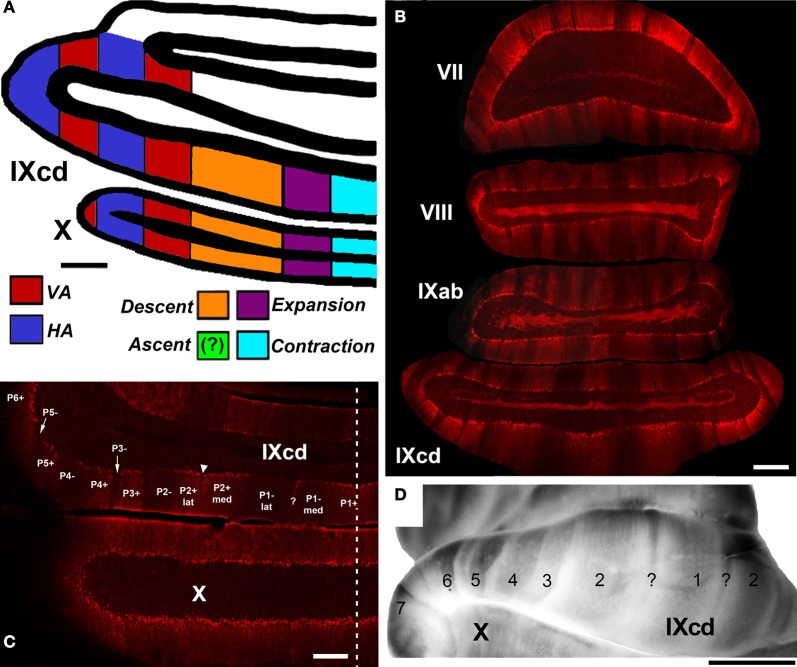
**(A)** Shows a schematic of the zonal organization of the optic flow responsive cells in the VbC as concluded from a series of electrophysiological and anatomical studies (Wylie and Frost, 1993, 1999b; Wylie et al., 1993, 2003a,b; Winship and Wylie, 2003; Pakan et al., 2005). The location of the *Ascent* units was unknown. **(B)** Shows a coronal section through the posterior cerebellum (folia VII–IXcd) showing heterogeneous zebrin II (ZII) expression. **(C)** and **(D)** Highlight the ZII expression in the vestibulocerebellum. **(D)** Is a wholemount of the cerebellum whereas **(C)** is a coronal section through folia IXcd and X. The ZII stripes are numbered P1± to P7± from the midline (indicated by the dashed line). P6−, P7+, and P7− are found more rostrally, as seen in **(D)**. P1− is divided into medial and lateral portions by a small satellite immunopositive band one to two Purkinje cells wide in the middle of P1− denoted “?”. P2+ is divided into medial and lateral portions by a small immunonegative “notch” in the middle of P2+ (see inverted triangle). Folium X does not have ZII stripes, as all Purkinje cells are ZII+ve. **(B)** and **(D)** Are adapted from Pakan et al. (2007). **(C)** Is adapted from Graham and Wylie (2012). Scale bars: **(A,B)** = 500 μm; **(C)** = 300 μm; **(D)** = 2.5 mm.

The functional units of the cerebellum are series of “zones” that lie in the sagittal plane, perpendicular to the axes of the folia. This organization is revealed in several aspects: afferents to the cerebellar cortex terminate in parasagittal bands (Voogd and Bigare, 1980; Wu et al., 1999; Ruigrok, 2003), and Purkinje cells within a sagittal band show similar response properties (Andersson and Oscarsson, 1978). As outlined by Simpson (2011), the flocculus is no exception in this regard, and this has been extensively studied in rabbits. Based on converging evidence examining the inferior olivary inputs to the flocculus, the projections of flocculus to the vestibular nuclei, eye movements elicited by stimulation of the flocculus, and the responses of Purkinje cells to rotational optic flow, it has been determined that there are four optic flow zones in the rabbit flocculus: two *VA* zones interdigitated with two *HA* zones (Kusunoki et al., 1990; DeZeeuw et al., 1994; Van der Steen et al., 1994; Tan et al., 1995). A strikingly similar organization has been found in pigeons: there are 2 *VA* zones interdigitated with 2 *HA* zones (Figure 8A). In other species it has also been shown that there is an interdigitation of *HA* and *VA* zones. In rats, there are 2 *HA* zones and 2 or 3 *VA* zones (Sugihara et al., 2004; Schonewille et al., 2006) whereas in macaques there appear to be two *VA* zone but only one *HA* zone (Voogd et al., 2012). Thus, the organization of optic rotational optic flow zones is highly conserved across birds and mammals (Voogd and Wylie, 2004).

This evolutionary conservation does not extend to the uvula/nodulus. The topographic organization of the zones in the uvula/nodulus of pigeons gleaned from several of our studies up until 2003 (Winship and Wylie, 2003; Wylie et al., 2003a,b) is shown in Figure 8A. In pigeons we showed that the *Contraction*, *Expansion* and *Descent* neurons were organized in three adjacent zones, from medial to lateral. We were uncertain as to the location of the *Ascent* neurons. In the mammalian uvula/nodulus, the *VA*, *HA*, and vestibular-responsive neurons are organized into parasaggital zones (Kano et al., 1990; Barmack and Shojaku, 1992).

## The relationship between the optic flow zones and zebrin stripes

A parasagittal organization is also seen in the cerebellum with respect to the expression of numerous molecular markers (Herrup and Kuemerle, 1997). The most thoroughly studied of these is zebrin II (ZII; the metabolic isoenzyme aldolase C), which is expressed almost exclusively by Purkinje cells (Brochu et al., 1990; Ahn et al., 1994; Hawkes and Herrup, 1995). ZII immunopositive (ZII+ve) Purkinje cells are distributed as a parasagittal array of stripes, separated by intervening ZII immunonegative (ZII−ve) stripes (Sillitoe et al., 2005; Larouche and Hawkes, 2006) (see Figure 8B). ZII stripes have been shown in several mammalian and avian species, with a strikingly similar pattern (Iwaniuk et al., 2009; Marzban and Hawkes, 2011). Thus, the pattern of ZII stripes is highly conserved, and likely critical for normal cerebellar function.

**Figure 9:**
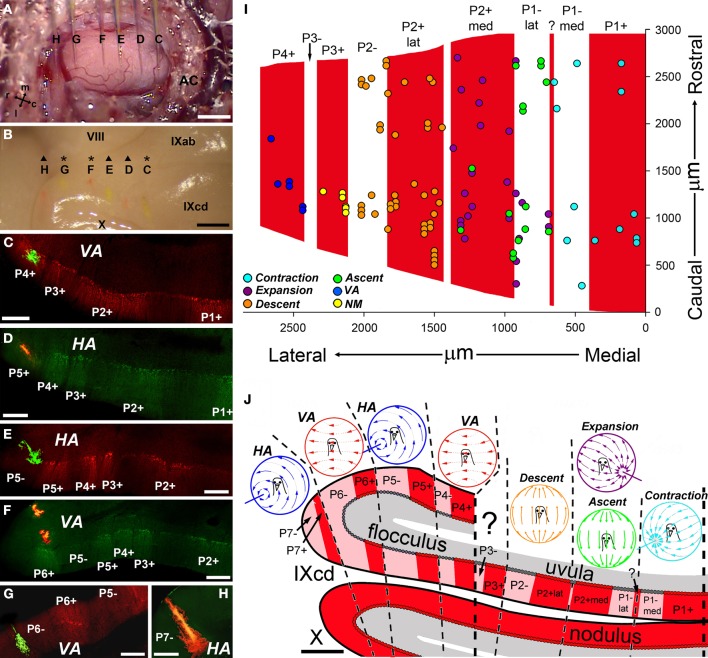
**Relationship between the optic flow zones and the zebrin II (ZII) stripes in the pigeon vestibulocerebellum (VbC). (A–H)** Show results from a single experiment. **(A)** Shows the surface of the exposed flocculus. This is the superimposition of six photos, such that the locations of six injection electrodes **(C–H)** filled with either red biotinylated dextran amine (BDA) **(D,F,H)** or green BDA **(C,E,G)** are shown. **(B)** Shows the subsequently perfused and dissected brain. Traces of the six injections can clearly be seen on the surface of IXcd. **(C–H)** Show coronal sections through the VbC that have been immunoreacted for ZII to illustrate the locations of all the injections in particular ZII stripes (from Pakan et al., 2011). **(I)** Shows our efforts to determine the locations of the translational optic flow zones relative to the ZII stripes in IXcd (Graham and Wylie, 2012). Collapsed from several recording experiments, the recording sites of *Contraction* (light blue), *Expansion* (purple), *Ascent* (green), and *Descent* (orange) cells are indicated, as well as some cells not modulated to visual stimuli (*NM*; yellow) and a few *VA* cells (dark blue). **(J)** Shows a summary of how the rotational and translational optic flow neurons are organized with respect to the ZII stripes. See text for additional details. ?: Small immunopositive satellite band one to two Purkinje cells wide in the middle of P1−; AC: anterior canal; m, l, r, and c: medial, lateral, rostral, and caudal. Scale bars: **(A,B)** = 1 mm; **(C–H)** = 300 μm; **(J)** = 500 μm.

The ZII stripes are apparent in folium IXcd of the pigeon VbC (Pakan et al., 2007; Figures 8B–D). However, in folium X there are no ZII stripes, as all the Purkinje cells are ZII+ve (Figures 8C and D). There are seven stripe pairs in IXcd numbered, from the midline, P1± to P7±. Importantly, the P1± stripe is divided into medial and lateral halves (P1− med, P1− lat) by a thin ZII+ stripe that is only 1–3 Purkinje cells wide (see “?” in Figures 8C and D). Also, the P2+ stripe is divided into medial and lateral halves (P2+ med, P2+ lat) by a thin ZII−ve notch (see inverted triangle in Figure 8C). In a recent series of studies (Pakan and Wylie, 2008; Pakan et al., 2011; Graham and Wylie, 2012), we have attempted to determine if the ZII stripes are correlated with the optic flow zones in the VbC. We found a clear relationship: each optic flow zone spans a ZII+ve/−ve stripe pair. Data regarding the floccular zones is shown in Figures 9A–H, from our most comprehensive case (Pakan et al., 2011). The procedure was to record from identified *HA* and *VA* neurons, mark the recording sites with an injection of red or green fluorescent tracer (biotinylated dextran amine; BDA), then subsequently process the tissue for ZII to determine the location of the recordings. Figure 9A shows the view of the flocculus through the surgical microscope with six injection pipettes superimposed. Those marked C, E, and G contained green BDA, whereas the others contained red BDA. When the perfused brain was dissected, the six injections could be clearly seen under a dissecting microscope (Figure 9B). At sites C, F, and G, *VA* neurons were recorded, whereas *HA* neurons were recorded at sites D, E, and H. As shown in the corresponding panels with the ZII expression pattern visualized in coronal sections, *VA* injections were localized to stripes P4+ (Figure 9C), P6+ (Figure 9F), and P6− (Figure 9G), whereas *HA* injections were found in stripes P5+ (Figure 9D), P5− (Figure 9E), and P7− (Figure 7H). Supplemented with data from other cases, we determined that the medial and lateral *VA* zones spanned the P4± and P6± stripe pairs, respectively, and the medial and lateral *HA* zones spanned the P5± and P7± stripe pairs, respectively (see Figure 9J). A similar story emerged for the translation optic flow zones in the uvula. Figure 9I shows the locations of identified neurons superimposed on the ZII stripes in IXcd from several cases (Graham and Wylie, 2012). The *Contraction* cells were localized to the P1+ and P1−med stripes, and the *Descent* cells were localized to the P2+lat and P2− stripes. *Ascent* and *Expansion* cells were found intermingled in the P2+med and P1−lat stripes. We did localize some cells to the P3+ stripe, but these were not modulated by the optic flow stimuli. The relationship between the ZII stripes and the optic flow zones in the VbC is summarized in Figure 9J. Each ZII+ve/−ve stripe pair spans an optic flow zone. Each one of these optic flow zones contains neurons with the same optic flow preference, with the exception of the one zone that contains both *Ascent* and *Expansion* neurons. Why this zone is peculiar in this regard is unknown, as is the function of the P3± stripe pair.

**Figure 10:**
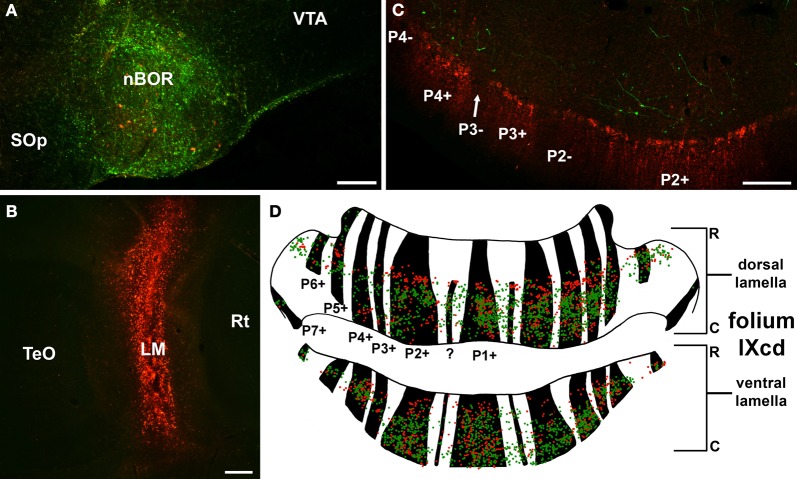
**Mossy fiber projections from the nucleus of the basal optic root (nBOR) and lentiformis mesencephali (LM) to the zebrin (ZII) stripes in folium IXcd. (A)** and **(B)** Show injections of green and red biotinylated dextran amine in nBOR and LM, respectively. **(C)** Shows a coronal section through IXcd reacted for ZII. The green terminal labeling from the nBOR is clustered adjacent to the ZII immunopositive (ZII+ve) stripes. **(D)** Shows a reconstruction of folium IXcd from serial sections. Each green and red dot represents a labeled terminal rosette from the injections in nBOR and LM, respectively, and the ZII+ve stripes are indicated in black. Note that most of the labeling is adjacent to the ZII+ve stripes. From Pakan et al. (2010). ?: Small immunopositive satellite band one to two Purkinje cells wide in the middle of P1−; Scale bars: **(A,B)** = 200 μm; **(C)** = 100 μm.

Although we have shown that each optic flow zone can be subdivided into a strip containing ZII+ve Purkinje cells and a strip containing ZII−ve Purkinje cells, the functional consequence of this remains unknown, as the function of ZII is not known. However, there are a few clues. First, shown in Figure 10, Pakan et al. (2010) found that most of the mossy fiber inputs from LM and nBOR (the green pathway shown in Figure 1) project adjacent to the ZII+ve stripes in IXcd. Thus, although both ZII+ve and ZII−ve neurons within a given optic flow zone receive visual input via climbing fibers from the mcIO, the ZII+ve cells seem to be getting more visual input via the mossy fiber pathways. Whether there are vestibular or somatomotor mossy afferents that project preferentially to ZII−ve stripes remains to be seen, but one could speculate that the ZII+ve and ZII−ve cells are processing different sensory information. Second, it has been shown that Purkinje cells in the ZII+ve and ZI−ve stripes within an optic flow zone likely project to different areas in the vestibular and cerebellar nuclei (Sugihara, 2011; Wylie et al., 2012). Finally, some studies have suggested that ZII+ve and ZII−ve cells may have different roles in plasticity (Nagao et al., 1997; Wadiche and Jahr, 2005; Ebner et al., 2012). For example, Paukert et al. (2010) showed that climbing fibers contacting ZII+ve Purkinje cells release more glutamate per action potential than those contacting ZII−ve Purkinje cells. They proposed that the ZII+ve Purkinje cells undergo more activity-dependent synaptic plasticity as a result. Thus, within an optic flow zone, there could be one system originating in ZII+ve stripes running in parallel with another system originating in ZII−ve stripes that differ with respect to: (1) mossy fiber inputs, (2) outputs to the vestibular and cerebellar nuclei, and (3) plasticity.

## Integration of local motion and optic flow in folia VI–VIII of the cerebellum

**Figure 11:**
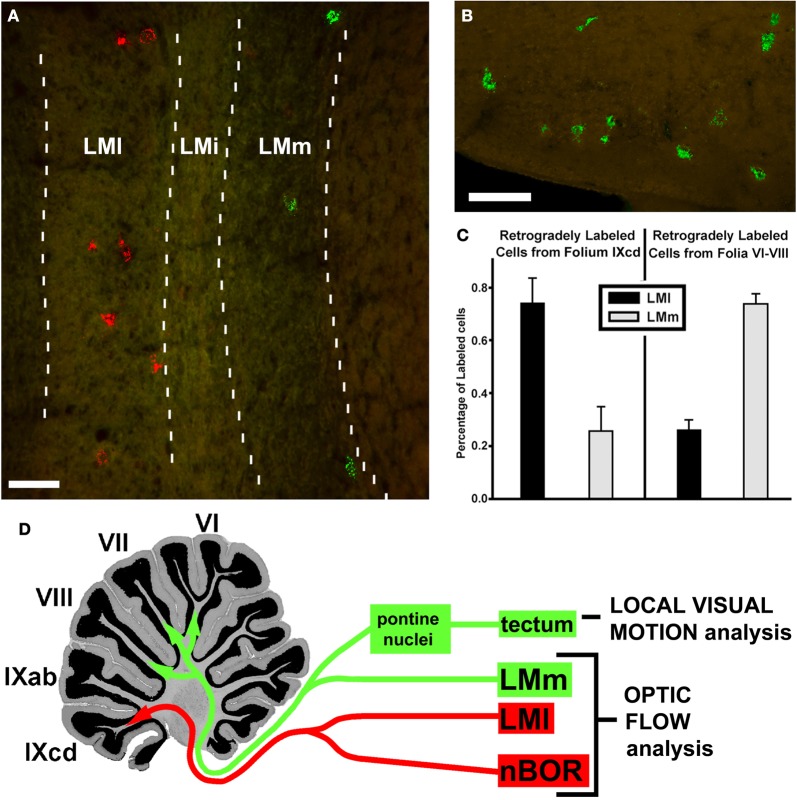
**Mossy fiber projections to the posterior cerebellum.** From an injection of green LumaFluor in folium VII, retrogradely labeled cells were found in the medial subnucleus of lentiformis mesencephali (LMm) **(A)** and the potine nuclei **(B)**. From an injection of red LumaFluor in folium IXcd, cells were labeled in nBOR (not shown) and the lateral subnucleus of LM **(A)**. **(C)** Shows the relative proportion of cells labeled in LMm (gray bars) and LMl (black bars) from injections in IXcd (left) and folia VI–VIII (right), collapsed from several cases. **(D)** Shows how in VI–VIII there is an integration of optic flow information, from LMm, with local motion information from a tecto-pontine system. From Pakan and Wylie (2006). LMi: intercalated subnucleus of LM. Scale bars: **(A,B)** = 100 μm.

In addition to the projection to IXcd, the LM also projects heavily to folia VI–VIII (Clarke, 1977), which is known as the oculomotor cerebellum (Voogd and Barmack, 2006). Pakan et al. (2006) investigated this projection using retrograde techniques. After injections of tracer into folia VI–VIII, most retrogradely labeled cells were found in LMm, whereas injections into IXcd labeled more cells in LMl (Figure 11). Injections into VI–VIII also labeled cells in the medial and lateral pontine nuclei. Previous reports have shown that the optic tectum projects to the pontine nuclei (Reiner and Karten, 1982). Thus, it appears that local motion from the tectum, and optic flow from LM may be integrated in the posterior cerebellum. What could be the function of this visual-visual integration? A few studies have shown that there is integration of local and optic flow information in primate visual cortex, and it has been suggested that this is important for “steering” to avoid obstacles during locomotion through cluttered environments (Page and Duffy, 2008; Elder et al., 2009). Hellmann et al. (2004) have suggested that the tecto-pontine pathway in birds is involved in avoidance behavior. Thus, perhaps the integration of optic flow and local motion signals in the posterior cerebellum of birds is important for obstacle avoidance as they fly through cluttered environments.

### Conflict of interest statement

The author declares that the research was conducted in the absence of any commercial or financial relationships that could be construed as a potential conflict of interest.

## References

[B1] Ahn A. H. Dziennis S. Hawkes R. Herrup K. (1994). The cloning of zebrin II reveals its identity with aldolase C. Development 120, 2081–2090 7925012 10.1242/dev.120.8.2081

[B2] Andersson G. Oscarsson O. (1978). Climbing fiber microzones in cerebellar vermis and their projection to different groups of cells in the lateral vestibular nucleus. Exp. Brain Res. 32, 565–579 689129 10.1007/BF00239553

[B3] Arends J. J. A. Voogd J. (1989). Topographic aspects of the olivocerebellar system in the pigeon. Exp. Brain Res. 17(Suppl.), 52–57

[B4] Barmack N. H. Shojaku H. (1992). Representation of a postural coordinate system in the nodulus of the rabbit cerebellum by vestibular climbing fiber signals, in Vestibular Control of Eye, Head and Body Movements, eds Shimazu H. Shinoda Y. (Tokyo; Japan Scientific Societies Press; Basel: Karger), 331–338

[B5] Brauth S. E. Karten H. J. (1977). Direct accessory optic projections to vestibulo-cerebellum– possible channel for oculomotor control-systems. Exp. Brain Res. 28, 73–84 881007 10.1007/BF00237087

[B6] Brecha N. Karten H. J. Hunt S. P. (1980). Projections of the nucleus of the basal optic root in the pigeon: an autoradiographic and horseradish peroxidase study. J. Comp. Neurol. 189, 615–670 10.1002/cne.901890404 7381044

[B7] Brochu G. Maler L. Hawkes R. (1990). Zebrin II: a polypeptide antigen expressed selectively by Purkinje cells reveals compartments in rat and fish cerebellum. J. Comp. Neurol. 291, 538–552 10.1002/cne.902910405 2329190

[B8] Burns S. Wallman J. (1981). Relation of single unit properties to the oculomotor function of the nucleus of the basal optic root (accessory optic system) in chickens. Exp. Brain Res. 42, 171–180 7262212 10.1007/BF00236903

[B9] Clarke P. G. (1977). Some visual and other connections to the cerebellum of the pigeon. J. Comp. Neurol. 174, 535–552 10.1002/cne.901740307 903417

[B10] Collewijn H. (1975). Direction-selective units in the rabbit's nucleus of the optic tract. Brain Res. 100, 489–508 10.1016/0006-8993(75)90154-7 172194

[B11] Crowder N. A. Dawson M. R. W. Wylie D. R. (2003). Temporal frequency and velocity-like tuning in the pigeon accessory optic system. J. Neurophysiol. 90, 1829–1841 10.1152/jn.00654.2002 12750415

[B12] Crowder N. A. Winship I. R. Wylie D. R. (2000). Topographic organization of inferior olive cells projecting to translational zones in the vestibulocerebellum of pigeons. J. Comp. Neurol. 419, 87–95 10.1002/(SICI)1096-9861(20000327)419:1<87::AID-CNE5>3.0.CO;2-W 10717641

[B12a] Crowder N. A. Wylie D. R. (2001). Fast and slow neurons in the nucleus of the basal optic root in pigeons. Neurosci. Lett. 304, 133–136 10.1016/S0304-3940(01)01734-7 11343820

[B13] DeZeeuw C. I. Wylie D. R. DiGiorgi P. L. Simpson J. I. (1994). Projections of individual Purkinje cells of physiologically identified zones in the flocculus to the vestibular and cerebellar nuclei in the rabbit. J. Comp. Neurol. 349, 428–447 10.1002/cne.903490308 7852634

[B14] Dickman J. D. Fang Q. (1996). Differential central projections of vestibular afferents in pigeons. J. Comp. Neurol. 367, 110–131 10.1002/(SICI)1096-9861(19960325)367:1<110::AID-CNE8>3.0.CO;2-6 8867286

[B15] Duffy C. J. (2004). The cortical analysis of optic flow, in The Visual Neurosciences, Vol. 2, eds Chalupa L. M. Werner J. S. (Cambridge, MA: MIT Press), 1260–1283

[B16] Duffy C. J. Wurtz R. H. (1991). Sensitivity of MST neurons to optic flow stimuli. I. A continuum of response selectivity to large-field stimuli. J. Neurophysiol. 65, 1329–1345 1875243 10.1152/jn.1991.65.6.1329

[B17] Ebner T. J. Wang X. Gao W. Cramer S. W. Chen G. (2012). Parasagittal zones in the cerebellar cortex differ in excitability, information processing, and synaptic plasticity. Cerebellum 11, 418–419 10.1007/s12311-011-0347-1 22249913 PMC3856581

[B18] Elder D. M. Grossberg S. Mingolla E. (2009). A neural model of visually guided steering, obstacle avoidance, and route selection. J. Exp. Psychol. Hum. Percept. Perform. 35, 1501–1531 10.1037/a0016459 19803653

[B19] Ezure K. Graf W. (1984). A quantitative analysis of the spatial organization of the vestibulo-ocular reflexes in lateral- and frontal-eyed animals: I. Orientation of semicircular canals and extraocular muscles. Neuroscience 12, 85–93 10.1016/0306-4522(84)90140-4 6611517

[B20] Fite K. V. (1985). Pretectal and accessory-optic visual nuclei of fish, amphibia and reptiles: themes and variations. Brain Behav. Evol. 26, 71–90 3907745 10.1159/000118769

[B21] Fite K. V. Brecha N. Karten H. J. Hunt S. P. (1981). Displaced ganglion cells and the accessory optic system of pigeon. J. Comp. Neurol. 195, 279–288 10.1002/cne.901950208 7251927

[B22] Frost B. J. Wylie D. R. (2000). A common frame of reference for the analysis of optic flow and vestibular information. Int. Rev. Neurobiol. 44, 121–140 10605644 10.1016/s0074-7742(08)60740-0

[B23] Gamlin P. D. R. (2005). The pretectum: connections and oculomotor-related roles. Prog. Brain Res. 151, 379–405 10.1016/S0079-6123(05)51012-4 16221595

[B24] Gamlin P. D. R. Cohen D. H. (1988). Projections of the retinorecipient pretectal nuclei in the pigeon (*Columba livia*). J. Comp. Neurol. 269, 1–17 3361002 10.1002/cne.902690103

[B25] Gibson J. J. (1954). The visual perception of objective motion and subjective movement. Psychol. Rev. 64, 304–314 13204493 10.1037/h0061885

[B26] Gioanni H. Rey J. Villalobos J. Richard D. Dalbera A. (1984). Single unit activity in the nucleus of the basal optic root (nBOR) during optokinetic, vestibular and visuo-vestibular stimulations in the alert pigeon (*Columba livia*). Exp. Brain Res. 57, 49–60 6335102 10.1007/BF00231131

[B27] Giolli R. A. Blanks R. H. Lui F. (2005). The accessory optic system: basic organization with an update on connectivity, neurochemistry, and function. Prog. Brain Res. 151, 407–440 10.1016/S0079-6123(05)51013-6 16221596

[B28] Graf W. Simpson J. I. Leonard C. S. (1988). Spatial organization of visual messages of the rabbit's cerebellar flocculus. II. Complex and simple spike responses of Purkinje cells. J. Neurophysiol. 60, 2091–2121 3236063 10.1152/jn.1988.60.6.2091

[B29] Graham D. J. Wylie D. R. (2012). Zebrin-immunopositive and -immunonegative stripe pairs represent functional units in the pigeon vestibulocerebellum. J. Neurosci. 32, 12769–12779 10.1523/JNEUROSCI.0197-12.2012 22973000 PMC6703799

[B30] Hawkes R. Herrup K. (1995). Aldolase C/zebrin II and the regionalization of the cerebellum. J. Mol. Neurosci. 6, 147–158 10.1007/BF02736761 8672398

[B31] Hellmann B. Gunturkun O. Manns M. (2004). Tectal mosaic: organization of the descending tectal projections in comparison to the ascending tectofugal pathway in the pigeon. J. Comp. Neurol. 472, 395–410 10.1002/cne.20056 15065115

[B32] Herrup K. Kuemerle B. (1997). The compartmentalization of the cerebellum. Annu. Rev. Neurosci. 20, 61–90 10.1146/annurev.neuro.20.1.61 9056708

[B33] Ibbotson M. R. Price N. S. (2001). Spatiotemporal tuning of directional neurons in mammalian and avian pretectum: a comparison of physiological properties. J. Neurophysiol. 86, 2621–2624 11698548 10.1152/jn.2001.86.5.2621

[B34] Iwaniuk A. N. Marzban H. Pakan J. M. Watanabe M. Hawkes R. Wylie D. R. (2009). Compartmentation of the cerebellar cortex of hummingbirds (Aves: Trochilidae) revealed by the expression of zebrin II and phospholipase C beta 4. J. Chem. Neuroanat. 37, 55–63 10.1016/j.jchemneu.2008.10.001 18996471

[B35] Kano M. Kano M. S. Kusunoki M. Maekawa K. (1990). Nature of optokinetic response and zonal organization of climbing fiber afferents in the vestibulocerebellum of the pigmented rabbit. II. The nodulus. Exp. Brain. Res. 80, 238–251 2358041 10.1007/BF00228152

[B36] Karten H. J. Fite K. V. Brecha N. (1977). Specific projection of displaced retinal ganglion cells upon the accessory optic system in the pigeon (*Columba livia*). Proc. Natl. Acad. Sci. U.S.A. 74, 1752–1756 266216 10.1073/pnas.74.4.1753 PMC430872

[B36a] Karten H. J. Shimizu T. (1991). Are visual hierarchies in the brains of the beholders?: Constancy and variability in the visual system of birds and mammals, in The Changing Visual System: Maturation and Aging in the Central Nervous System, eds Bagnoli P. Hodos W. (New York, NY: Plenum Press), 51–59

[B37] Kearns M. J. Warren W. H. Duchon A. P. Tarr M. J. (2002). Path integration from optic flow and body senses in a homing task. Perception 31, 349–374 11954696 10.1068/p3311

[B38] Krapp H. G. Hengstenberg R. (1996). Estimation of self-motion by optic flow processing in single visual interneurons. Nature 384, 463–466 10.1038/384463a0 8945473

[B39] Krapp H. G. Hengstenberg B. Hengstenberg R. (1998). Dendritic structure and receptive-field organization of optic flow processing interneurons in the fly. J. Neurophysiol. 79, 1902–1917 9535957 10.1152/jn.1998.79.4.1902

[B40] Kusunoki M. Kano M. Kano M. S. Maekawa K. (1990). Nature of optokinetic response and zonal organization of climbing fiber afferents in the vestibulocerebellum of the pigmented rabbit. I. The flocculus. Exp. Brain Res. 80, 225–237 2358040 10.1007/BF00228151

[B41] Larouche M. Hawkes R. (2006). From clusters to stripes: the developmental origins of adult cerebellar compartmentation. Cerebellum 5, 77–88 10.1080/14734220600804668 16818382

[B42] Larsell O. (1967). The Cerebellum: From Myxinoids Through Birds. Minneapolis, MN: The University of Minnesota Press

[B43] Lau K. L. Glover R. G. Linkenhoker B. Wylie D. R. (1998). Topographical organization of inferior olive cells projecting to translation and rotation zones in the vestibulocerebellum of pigeons. Neuroscience 85, 605–614 10.1016/S0306-4522(97)00620-9 9622256

[B44] Marzban H. Hawkes R. (2011). On the architecture of the posterior zone of the cerebellum. Cerebellum 10, 422–434 10.1007/s12311-010-0208-3 20838950

[B45] McKenna O. C. Wallman J. (1985). Accessory optic system and pretectum of birds: comparisons with those of other vertebrates. Brain Behav. Evol. 26, 91–116 3907746 10.1159/000118770

[B46] Morgan B. Frost B. J. (1981). Visual response characteristics of neurons in nucleus of the basal optic root of pigeons. Exp. Brain Res. 42, 181–188 7262213 10.1007/BF00236904

[B47] Nagao S. Kitazawa H. Osanai R. Hiramatsu T. (1997). Acute effects of tetrahydrobiopterin on the dynamic characteristics and adaptability of vestibulo-ocular reflex in normal and flocculus lesioned rabbits. Neurosci. Lett. 231, 41–44 10.1016/S0304-3940(97)00518-1 9280163

[B48] Nguyen A. P. Spetch M. L. Crowder N. C. Winship I. R. Hurd P. L. Wylie D. R. (2004). A dissociation of motion and spatial-pattern vision in the avian telencephalon: implications for the evolution of “visual streams.” J. Neurosci. 24, 4962–4970 10.1523/JNEUROSCI.0146-04.2004 15163688 PMC6729365

[B49] Page W. K. Duffy C. J. (2008). Cortical neuronal responses to optic flow are shaped by visual strategies for steering. Cereb. Cortex 18, 727–739 10.1093/cercor/bhm109 17621608

[B50] Pakan J. M. P. Graham D. J. Gutiérrez-Ibáñez C. Wylie D. R. (2011). Organization of the cerebellum: correlating zebrin immunochemistry with optic flow zones in the pigeon flocculus. Vis. Neurosci. 28, 163–174 10.1017/S0952523810000532 21463542

[B51] Pakan J. M. P. Graham D. J. Iwaniuk A. N. Wylie D. R. (2008). Differential projections from the vestibular nuclei to the flocculus and uvula-nodulus in pigeons (*Columba livia*). J. Comp. Neurol. 508, 402–417 10.1002/cne.21623 18335537

[B52] Pakan J. M. P. Graham D. J. Wylie D. R. (2010). Organization of visual mossy fiber projections and zebrin expression in the pigeon vestibulocerebellum. J. Comp. Neurol. 518, 175–198 10.1002/cne.22192 19937710

[B53] Pakan J. M. P. Iwaniuk A. N. Wylie D. R. Hawkes R. Marzban H. (2007). Purkinje cell compartmentation as revealed by zebrin II expression in the cerebellar cortex of pigeons (*Columba livia*). J. Comp. Neurol. 501, 619–630 10.1002/cne.21266 17278140

[B54] Pakan J. M. P. Todd K. G. Krueger K. Kelcher E. Cooper S. Wylie D. R. (2006). Projections of the nucleus lentiformis mesencephali in pigeons (*Columba livia*): a comparison of the morphology and distribution of neurons with different efferent projections. J. Comp. Neurol. 495, 84–99 10.1002/cne.20855 16432900

[B55] Pakan J. M. P. Todd K. G. Ngyuen A. P. Winship I. R. Hurd P. L. Jantzie L. (2005). Inferior olivary neurons innervate multiple zones of the flocculus in pigeons (*Columba livia*). J. Comp. Neurol. 486, 159–168 10.1002/cne.20523 15844212

[B56] Pakan J. M. P. Wylie D. R. W. (2006). Two optic flow pathways from the pretectal nucleus lentiformis mesencephali to the cerebellum in pigeons (*Columba livia*). J. Comp. Neurol. 499, 732–744 10.1002/cne.21108 17048227

[B57] Pakan J. M. P. Wylie D. R. (2008). Congruence of zebrin II expression and functional zones defined by climbing fibre topography in the flocculus. Neuroscience 157, 57–69 10.1016/j.neuroscience.2008.08.062 18824220

[B58] Paukert M. Huang Y. H. Tanaka K. Rothstein J. D. Bergles D. E. (2010). Zones of enhanced glutamate release from climbing fibers in the mammalian cerebellum. J. Neurosci. 30, 7290–7299 10.1523/JNEUROSCI.5118-09.2010 20505095 PMC2894469

[B59] Reiner A. Brecha N. Karten H. J. (1979). A specific projection of retinal displaced ganglion cells to the nucleus of the basal optic root in the chicken. Neuroscience 4, 1679–1688 92770 10.1016/0306-4522(79)90027-7

[B60] Reiner A. Karten H. J. (1982). Laminar distribution of the cells of origin of the descending tectofugal pathways in the pigeon (*Columba livia*). J. Comp. Neurol. 204, 165–187 10.1002/cne.902040206 7056890

[B61] Ruigrok T. J. (2003). Collateralization of climbing and mossy fibers projecting to the nodulus and flocculus of the rat cerebellum. J. Comp. Neurol. 466, 278–298 10.1002/cne.10889 14528453

[B62] Schwarz I. E. Schwarz D. W. (1983). The primary vestibular projection to the cerebellar cortex in the pigeon (*Columba livia*). J. Comp. Neurol. 216, 438–444 10.1002/cne.902160409 6308074

[B63] Schonewille M. Luo C. Ruigrok T. J. Voogd J. Schmolesky M. T. Rutteman M. (2006). Zonal organization of the mouse flocculus: physiology, input, and output. J. Comp. Neurol. 497, 670–682 10.1002/cne.21036 16739198

[B64] Sillitoe R. V. Marzban H. Larouche M. Zahedi S. Affanni J. Hawkes R. (2005). Conservation of the architecture of the anterior lobe vermis of the cerebellum across mammalian species. Prog. Brain Res. 148, 283–297 10.1016/S0079-6123(04)48022-4 15661197

[B65] Simpson J. I. (1984). The accessory optic system. Ann. Rev. Neurosci. 7, 13–41 10.1146/annurev.ne.07.030184.000305 6370078

[B66] Simpson J. I. (2011). Crossing zones in the vestibulocerebellum: a commentary. Cerebellum 10, 515–522 10.1007/s12311-011-0305-y 21822546

[B67] Simpson J. I. Alley K. E. (1974). Visual climbing fiber input to rabbit vestibulo-cerebellum: a source of direction-specific information. Brain Res. 82, 302–308 10.1016/0006-8993(74)90610-6 4441896

[B68] Simpson J. I. Graf W. (1985). The selection of reference frames by nature and its investigators, in Adaptive Mechanisms in Gaze Control: Facts and Theories, eds Berthoz A. Melvill-Jones G. (Amsterdam: Elsevier), 3–16 3940037

[B69] Simpson J. I. Graf W. Leonard C. (1981). The coordinate system of visual climbing fibres to the flocculus, in Progress in Oculomotor Research, eds Fuchs A. F. Becker W. (Amsterdam: Elsevier), 475–484

[B70] Simpson J. I. Leonard C. S. Soodak R. E. (1988). The accessory optic system. II. Spatial organization of direction selectivity. J. Neurophysiol. 60, 2055–2072 3236061 10.1152/jn.1988.60.6.2055

[B71] Simpson J. I. Soodak R. E. Hess R. (1979). The accessory optic system and its relation to the vestibulocerebellum. Prog. Brain Res. 50, 715–724 10.1016/S0079-6123(08)60868-7 551466

[B72] Srinivasan M. Zhang S. Lehrer M. Collett T. (1996). Honeybee navigation en route to the goal: visual flight control and odometry. J. Exp. Biol. 199, 237–244 9317712 10.1242/jeb.199.1.237

[B73] Sugihara I. (2011). Compartmentalization of the deep cerebellar nuclei based on afferent projections and aldolase C expression. Cerebellum 10, 449–463 10.1007/s12311-010-0226-1 20981512

[B74] Sugihara I. Ebata S. Shinoda Y. (2004). Functional compartmentalization in the flocculus and the ventral dentate and dorsal group y nuclei: an analysis of single olivocerebellar axonal morphology. J. Comp. Neurol. 470, 113–133 10.1002/cne.10952 14750156

[B75] Tan J. Gerrits N. M. Nanhoe R. Simpson J. I. Voogd J. (1995). Zonal organization of the climbing fiber projection to the flocculus and nodulus of the rabbit: a combined axonal tracing and acetylcholinesterase histochemical study. J. Comp. Neurol. 356, 23–50 10.1002/cne.903560103 7543121

[B76] Tanaka K. Fukada Y. Saito H. A. (1989). Underlying mechanisms of the response specificity of expansion/contraction and rotation cells in the dorsal part of the medial superior temporal area of the macaque monkey. J. Neurophysiol. 62, 642–656 2769352 10.1152/jn.1989.62.3.642

[B77] Tanaka K. Saito H. (1989). Analysis of motion of the visual field by direction, expansion/contraction, and rotation cells clustered in the dorsal part of the medial superior temporal area of the macaque monkey. J. Neurophysiol. 62, 626–641 2769351 10.1152/jn.1989.62.3.626

[B78] Van der Steen J. Simpson J. I. Tan J. (1994). Functional and anatomic organization of three-dimensional eye movements in rabbit cerebellar flocculus. J. Neurophysiol. 72, 31–46 7965015 10.1152/jn.1994.72.1.31

[B79] Voogd J. Barmack N. H. (2006). Oculomotor cerebellum. Prog. Brain Res. 151, 231–268 10.1016/S0079-6123(05)51008-2 16221591

[B80] Voogd J. Bigare F. (1980). Topographical distribution of olivary and corticonuclear fibres in the cerebellum: a review, in The Olivary Nucleus. Anatomy and Physiology, eds de Montigny C. Courville J. (New York, NY: Raven), 207–234

[B81] Voogd J. Schraa-Tam C. K. van der Geest J. N. De Zeeuw C. I. (2012). Visuomotor cerebellum in human and nonhuman primates. Cerebellum 11, 392–410 10.1007/s12311-010-0204-7 20809106 PMC3359447

[B82] Voogd J. Wylie D. R. (2004). Functional and anatomical organization of floccular zones: a preserved feature in vertebrates. J. Comp. Neurol. 470, 107–112 10.1002/cne.11022 14750155

[B83] Wadiche J. I. Jahr C. E. (2005). Patterned expression of Purkinje cell glutamate transporters controls synaptic plasticity. Nat. Neurosci. 8, 1329–1334 10.1038/nn1539 16136036

[B84] Waespe W. Henn V. (1987). Gaze stabilization in the primate. The interaction of the vestibulo-ocular reflex, optokinetic nystagmus, and smooth pursuit. Rev. Physiol. Biochem. Pharmacol. 106, 37–125 3303269

[B85] Warren W. H. Jr. Kay B. A. Zosh W. D. Duchon A. P. Sahuc S. (2001). Optic flow is used to control human walking. Nat. Neurosci. 4, 213–216 10.1038/84054 11175884

[B86] Weber J. T. (1985). Pretectal complex and accessory optic system in alert monkeys. Brain Behav. Evol. 26, 117–140 3907744 10.1159/000118771

[B87] Wild J. M. (1989). Pretectal and tectal projections to the homologue of the dorsal lateral geniculate nucleus in the pigeon: an anterograde and retrograde tracing study with choleratoxin conjugated to horseradish peroxidase. Brain Res. 479, 130–137 10.1016/0006-8993(89)91342-5 2924142

[B88] Wilson J. Melvill Jones G. (1979). Mammalian Vestibular Physiology. New York NY: Plenum Press

[B89] Winship I. R. Pakan J. M. P. Todd K. G. Wong-Wylie D. R. (2006). A comparison of ventral tegmental neurons projecting to optic flow regions of the inferior olive vs. the hippocampal formation. Neuroscience 141, 463–473 10.1016/j.neuroscience.2006.03.057 16698184

[B90] Winship I. R. Wylie D. R. (2006). Receptive field structure of optic flow responsive Purkinje cells in the vestibulocerebellum of pigeons. Vis. Neurosci. 23, 115–126 10.1017/S0952523806231109 16597355

[B91] Winship I. R. Wylie D. R. (2003). Zonal organization of the vestibulocerebellum in pigeons (*Columba livia*): I. Climbing fibre input to the flocculus. J. Comp. Neurol. 456, 127–139 10.1002/cne.10507 12509870

[B92] Winship I. R. Wylie D. R. (2001). Responses of neurons in the medial column of the inferior olive in pigeons to translational and rotational optic flowfields. Exp. Brain Res. 141, 63–78 10.1007/s002210100845 11685411

[B93] Winterson B. J. Brauth S. E. (1985). Direction-selective single units in the nucleus lentiformis mesencephali of the pigeon (*Columba livia*). Exp. Brain Res. 60, 215–226 4054266 10.1007/BF00235916

[B94] Wu H. S. Sugihara I. Shinoda Y. (1999). Projection patterns of single mossy fibres originating from the lateral reticular nucleus in the rat cerebellar cortex and nuclei. J. Comp. Neurol. 411, 97–118 10.1002/(SICI)1096-9861(19990816)411:1<97::AID-CNE8>3.0.CO;2-O 10404110

[B95] Wylie D. R. (2001). Projections from the nucleus of the basal optic root and nucleus lentiformis mesencephali to the inferior olive in pigeons (*Columba livia*). J. Comp. Neurol. 429, 502–513 10.1002/1096-9861(20010115)429:3<502::AID-CNE10>3.0.CO;2-E 11116234

[B96] Wylie D. R. (2000). Binocular neurons in the nucleus lentiformis mesencephali in pigeons: responses to translational and rotational optic flowfields. Neurosci. Lett. 291, 9–12 10.1016/S0304-3940(00)01367-7 10962141

[B97] Wylie D. R. Bischof W. F. Frost B. J. (1998a). Common reference frame for coding translational and rotational optic flow. Nature 392, 278–282 10.1038/32648 9521321

[B98] Wylie D. R. Glover R. G. Lau K. L. (1998b). Projections from the accessory optic system and pretectum to the dorsolateral thalamus in the pigeon (*Columba livia*): a study using both anterograde and retrograde tracers. J. Comp. Neurol. 391, 456–469 10.1002/(SICI)1096-9861(19980222)391:4<456::AID-CNE4>3.0.CO;2-# 9486825

[B99] Wylie D. R. Brown M. R. Barkley R. R. Winship I. R. Todd K. G. (2003a). Zonal organization of the vestibulocerebellum in pigeons (*Columba livia*): II. projections of the rotation zones of the flocculus. J. Comp. Neurol. 456, 140–153 10.1002/cne.10508 12509871

[B100] Wylie D. R. Brown M. R. Winship I. R. Crowder N. A. Barkley R. R. Todd K. G. (2003b). Zonal organization of the vestibulocerebellum in pigeons (*Columba livia*): III. projections of the translation zones of the ventral uvula and nodulus. J. Comp. Neurol. 465, 179–194 10.1002/cne.10857 12949780

[B101] Wylie D. R. Crowder N. A. (2000). Spatio-temporal properties of “fast” and “slow” direction-selective neurons in the pretectal nucleus lentiformis mesencephali in pigeons. J. Neurophysiol. 84, 2529–2540 11067995 10.1152/jn.2000.84.5.2529

[B102] Wylie D. R. De Zeeuw C. I. DiGiorgi P. L. Simpson J. I. (1994). Projections of individual Purkinje cells of physiologically identified zones in the ventral nodulus to the vestibular and cerebellar nuclei in the rabbit. J. Comp. Neurol. 349, 448–463 10.1002/cne.903490309 7852635

[B103] Wylie D. R. Frost B. J. (1990a). Visual response properties of neurons in the nucleus of the basal optic root of the pigeon: a quantitative analysis. Exp. Brain Res. 82, 327–336 2286235 10.1007/BF00231252

[B104] Wylie D. R. Frost B. J. (1990b). Binocular neurons in the nucleus of the basal optic root (nBOR) of the pigeon are selective for either translational or rotational visual flow. Vis. Neurosci. 5, 489–495 2288897 10.1017/s0952523800000614

[B105] Wylie D. R. Frost B. J. (1991). Purkinje cells in the vestibulocerebellum of the pigeon respond best to either translational or rotational visual flow. Exp. Brain Res. 86, 229–232 1756795 10.1007/BF00231059

[B106] Wylie D. R. Frost B. J. (1993). Responses of pigeon vestibulocerebellar neurons to optokinetic stimulation: II. The 3-dimensional reference frame of rotation neurons in the flocculus. J. Neurophysiol. 70, 2647–2659 8120604 10.1152/jn.1993.70.6.2647

[B107] Wylie D. R. Frost B. J. (1996). The pigeon optokinetic system: visual input in extraocular muscle coordinates. Vis. Neurosci. 13, 945–953 8903035 10.1017/s0952523800009172

[B108] Wylie D. R. Frost B. J. (1999a). Complex spike activity of Purkinje cells in the ventral uvula and nodulus of pigeons in response to translational optic flowfields. J. Neurophysiol. 81, 256–266 9914286 10.1152/jn.1999.81.1.256

[B109] Wylie D. R. Frost B. J. (1999b). Responses of neurons in the nucleus of the basal optic root to translational and rotational optic flowfields. J. Neurophysiol. 81, 267–276 9914287 10.1152/jn.1999.81.1.267

[B110] Wylie D. R. Kripalani T.-K. Frost B. J. (1993). Responses of pigeon vestibulocerebellar neurons to optokinetic stimulation: I. functional organization of neurons discriminating between translational and rotational visual flow. J. Neurophysiol. 70, 2632–2646 8120603 10.1152/jn.1993.70.6.2632

[B111] Wylie D. R. Linkenhoker B. (1996). Mossy fibres from the nucleus of the basal optic root project to the vestibular and cerebellar nuclei in pigeons. Neurosci. Lett. 219, 83–86 10.1016/S0304-3940(96)13176-1 8971785

[B112] Wylie D. R. Linkenhoker B. Lau K. L. (1997). Projections of the nucleus of the basal optic root in pigeons (*Columba livia*) revealed using biotinylated dextran amine. J. Comp. Neurol. 384, 517–536 10.1002/(SICI)1096-9861(19970811)384:4<517::AID-CNE3>3.0.CO;2-5 9259487

[B113] Wylie D. R. Pakan J. M. P. Elliott C. A. Graham D. J. Iwaniuk A. N. (2007). Projections of the nucleus of the basal optic root in pigeons (*Columba livia*): a comparison of the morphology and distribution of neurons with different efferent projections. Vis. Neurosci. 24, 691–707 10.1017/S0952523807070599 17915041

[B114] Wylie D. R. Pakan J. M. P. Gutiérrez-Ibáñez C. Iwaniuk A. N. (2008). Expression of calcium binding proteins in pathways from the nucleus of the basal optic root to the cerebellum in pigeons. Vis. Neurosci. 25, 701–707 19112657 10.1017/s0952523808080772

[B115] Wylie D. R. Pakan J. M. P. Huynh H. Graham D. J. Iwaniuk A. N. (2012). The distribution of zebrin immunoreactive Purkinje cell terminals in the cerebellar and vestibular nuclei of birds. J. Comp. Neurol. 520, 1532–1546 10.1002/cne.22810 22105608

[B116] Wylie D. R. W. Winship I. Glover R. G. (1999). Projections from the medial column of the inferior olive to different classes of rotation-sensitive Purkinje cells in the flocculus of pigeons. Neurosci. Lett. 268, 97–100 10.1016/S0304-3940(99)00390-0 10400087

[B117] Yakusheva T. Blazquez P. M. Angelaki D. E. (2008). Frequency-selective coding of translation and tilt in macaque cerebellar nodulus and uvula. J. Neurosci. 28, 9997–10009 10.1523/JNEUROSCI.2232-08.2008 18829957 PMC2586807

